# The Role of Attitudes, Affect, and Income in Predicting COVID-19 Behavioral Intentions

**DOI:** 10.3389/fpsyg.2020.567397

**Published:** 2021-01-06

**Authors:** Kelly S. Clemens, John Matkovic, Kate Faasse, Andrew L. Geers

**Affiliations:** ^1^Department of Psychology, University of Toledo, Toledo, OH, United States; ^2^School of Population Health, University of Toledo, Toledo, OH, United States; ^3^School of Psychology, University of New South Wales, Kensington, NSW, Australia

**Keywords:** COVID-19, behavioral intentions, handwashing, readiness to change, affective responses, emotion, attitudes

## Abstract

Handwashing is important in preventing infectious diseases like COVID-19. The current public health emergency has required rapid implementation of increased handwashing in the general public; however, rapidly changing health behavior, especially on this scale, is difficult. This study considers attitudes and affective responses to handwashing as possible factors predicting COVID-19 related changes to handwashing behavior, future intentions, and readiness to change during the early stages of the pandemic in the United States. Income was explored as a potential moderator to these relationships. To explore these issues, data from 344 community participants were analyzed. Results indicate that stronger affective responses toward handwashing relate to increases in handwashing since the outbreak of COVID-19, and both attitudes and affect uniquely predict handwashing intentions. Income significantly moderated the relationship between affect and readiness to change. Those with low income were more influenced by both affective responses and attitudes. These results suggest messages targeting both cognitions and affective responses are needed to increase the handwashing behavior during a global pandemic and these variables are critical in increasing readiness to change in low-income individuals.

## Introduction

Due to the rapid spread of the SARS-CoV-2 virus, it was critical that Americans quickly implemented health behaviors such as social distancing and frequent handwashing. This implementation is, perhaps, easier said than done, as rapid implementation of behavior change is notoriously difficult to achieve in many health domains. With the backdrop of a global pandemic, it was unclear how Americans would response to this need for behavior change, or what factors may be influential. Handwashing was not only among the first behaviors recommended to the public, remaining visible to the public throughout the course of the pandemic, but it is also central to the prevention of the spread of SARS-CoV-2 (West et al., 2020). As such, it is logical to explore it as a target behavior. The present study builds on emerging literature (Williams et al., 2018) and hypothesizes that affective and cognitive variables are critical and distinct predictors of behavior, behavioral intentions, and readiness for behavior change. Further, as disparities have been seen in the potential risk to those who have lower incomes (Koma et al., 2020; Raifman and Raifman, 2020), income will be explored as a potential moderator.

Factors related to behavioral intentions and change have been widely explored, however the rapidity of the change due to the pandemic in the United States is unprecedented, and it is unclear what factors will be most influential on behavior and intentions. Cognitive variables, such as attitudes, have long been established as consistent predictors of behavioral intentions in models such as the Theory of Planned Behavior (Ajzen, 1991), and attitudes have previously been found to significantly predict prevention-related behavioral intentions related to the Ebola epidemic (Gamma et al., 2017). Determining behavioral intentions, however, may give an incomplete picture of actual behavior. Readiness to make behavioral changes may help to fill this gap, as it has also been found to be uniquely predictive of the actual behavior in other domains (Biller et al., 2000; Geller et al., 2004). Attitudes have also been found to predict individuals’ temporal readiness to change, or stage of change (Ronda et al., 2001). The present study seeks to confirm that attitudes continue to predict behavior, intentions, and readiness to change during a rapidly moving pandemic with diverse and intense health-related messaging and personal relevance.

Alongside attitudes, affective responses to health behaviors have recently been acknowledged as important predictors of health behaviors (Schuettler and Kiviniemi, 2006; Rhodes et al., 2009; Kwan and Bryan, 2010; Ferrer et al., 2016). For example, the Behavioral Affective Association Model (BAAM; Kiviniemi and Klasko-Foster, 2018) contends that affective associations with health behavior are a critical and often underappreciated determinant of action. The BAAM identifies cognitive variables (e.g., perceived norms) and affective variables (e.g., positive feelings) as independent predictors of intentions and health behaviors (e.g., Brown-Kramer and Kiviniemi, 2015). In line with such models, studies indicate that affective variables can be separate predictors of health actions and intentions from cognitive variables, such as attitudes (Lowe et al., 2002; Kiviniemi et al., 2007; Conner et al., 2013; Geers et al., 2017). For example, using simultaneous regression analyses, Lawton et al. (2009) found that affective and instrumental attitudes were separate significant predictors of intentions to perform 11 different health behaviors, including alcohol consumption, flossing, and sunscreen use. Similarly, Murray et al. (2019) recently found that affective associations with physical activity and perceived barriers to physical activity were separate and simultaneous predictors of physical activity in cancer survivors. Interestingly, in these studies, the affect variables were generally a stronger predictor of health intentions and behavior than the cognitive variables. It now appears that behavior change interventions can be optimized if they specifically target changing emotions as well as attitudes (for a review, see Williams et al., 2018). Based on this emerging database, it is predicted in the present study that affective responses to handwashing will be distinctly predictive of behavior and intentions. While there is little evidence to date on the impact of affective responses on the stages of change, affect is inherently tied to the processes of change (e.g., dramatic relief; Prochaska et al., 2008), and thus it is also predicted that affective response will be uniquely predictive of readiness to change handwashing behavior during the early stages of a global pandemic.

While attitudes and affect are expected to drive handwashing behavior, intentions, and readiness to change, it is possible that the influence of these predictors varies with important social factors. One possibility, explored here, is that income moderates these effects. It is well documented that income and factors related to income are determinants of health (USDDS, 2020). Emerging data suggests that monetarily impoverished communities are most impacted by COVID-19. A recent analysis of the Behavioral Risk Factor Surveillance System data by Koma et al. (2020) from the Centers for Disease Control and Prevention (CDC) revealed that lower income was associated with more risk factors for becoming seriously ill with COVID-19 in the United States (also see Raifman and Raifman, 2020). In addition to low-income being associated with risk factors related to COVID-19 such as diabetes (Bird et al., 2015), chronic kidney disease (Nicholas et al., 2015), and heart disease (Lemstra et al., 2015), low income may also increase risk due to behavior. Those living in low-income areas have been found to be less likely to follow stay home directives (Chiou and Tucker, 2020), perhaps due to the lack of time off, job requirements, or being unable to forego income. Furthermore, income plays a particularly strong role in perceptions of information about healthy behaviors in the United States: Those with high income are more positive about their health and health care than those with low income (Hero et al., 2017). As low income can relate to riskier behavior and less responsiveness to health care information, positive changes in affective responses and attitudes for these individuals may result in greater benefits. Understanding the impact of income on the processes behind key health behaviors would shed light on psychological factors involved in the different paths of low- and high-income individuals and may highlight directions for more targeted intervention. As such, income will be explored as a moderator of the relationship between both attitudes and affect and the dependent variables of handwashing behavior, intentions, and readiness to change. It should be noted that there is some overlap in the data presented in the current study and a paper by Matkovic et al. (2020), including demographic items, handwashing attitudes, affect, intentions, and readiness to change. However, while the study by Matkovic and colleagues focused on differences in variables in response to a message intervention manipulation, this study focused on modeling the relationships among variables including attitudes, affect, and intentions, in order to better understand the pathways these variables take in affecting behavior. While there is an overlap in the data used, separate *a priori* hypotheses were made and separate analyses were run for each study.

## Materials and Methods

### Participants

Community participants (*N* = 344) were recruited from Prolific, an online participant recruitment system at the end of March 2020, just as stay home and shelter in place orders began to be issued in many states. Participants ranged in age from 18 to 74 (*M* = 32.69, *SD* = 11.60), and were fairly evenly distributed by gender, with 54.1% identifying as women, 43.9% identifying as men, and 1.5% identifying as another gender or preferring not to disclose their gender. Participants lived in the United States at the time of survey completion and represented 44 of the states. Participants were 68% White, 16% Asian/Asian-American, 5% Black, 5% Latinx, and 6% two or more races. Participants were compensated for participating in the study.

### Measures

Participants completed measures through the Qualtrics survey platform and provided information about their handwashing attitudes, affective responses, intentions, and readiness to change.

#### Demographic Items

Demographic items, including age, gender, race and ethnicity, and household income, were included at the end of the study. A series of items relating to geographic location, cases of COVID-19 occurring in near proximity, and risk factors related to COVID-19 were also collected at this time. Of interest in the present study, household income was collected as a multiple-choice item in which participants indicated their household income in $9,999 increments. Eleven options were provided, with the final choice indicating “$100,000 or more.” Of the respondents, 52.6% fell in the income range of $20,000 and $79,999, and 19.80% reported household incomes $100,000 or more.

#### Handwashing Attitudes

Handwashing attitudes were assessed using two items presented on a 7-point Likert scale ranging from *strongly agree* to *strongly disagree*. The items asked participants to rate how important and effective handwashing is in preventing disease, reading “Handwashing is effective in preventing disease” and “Handwashing is important.” The two items showed acceptable internal consistency with a Spearman-Brown coefficient of 0.78, and were averaged together to create a single bipolar attitude toward handwashing measure, ranging from negative to positive.

#### Handwashing Affect

Five items that assessed affective responses related to handwashing were measured in order to capture the strength of these affective responses. Items included both positively and negatively valanced affective states, including anger, pride, guilt, annoyance, and feeling in control. Example items include “I am angry when others do not wash their hands” and “I am proud of washing my hands.” Items were scored on a 7-point Likert scale ranging from *strongly disagree* to *strongly agree*. The five items had a high level of internal consistency (*α* = 0.83). As such, the five items were averaged together to create a single bipolar affect toward handwashing measure.

#### COVID-19 Handwashing Behavior Change

COVID-19 related handwashing behavior change was calculated by asking participants to self-report the number to times they washed their hands daily before the outbreak of COVID-19 and then after the outbreak of COVID-19. Both items allowed participants to enter a numeric response into an open response item. Analyses were conducted on the post-COVID-19 handwashing behavior while controlling for self-reported pre-COVID-19 handwashing behavior.

#### Handwashing Intentions

Handwashing intentions were assessed using six questions that targeted the intention to wash one’s hands in scenarios recommended by health organizations, such as “after blowing your nose, coughing or sneezing” and “after touching surfaces outside of the home, including money.” The final item asked about intention to wash one’s hands for at least 20 s each time. The scale demonstrated a high level of internal consistency (*α* = 0.80).

#### Readiness to Change

Temporal readiness to change, or the stage of change that a participant is in, was simply assessed with a single item modeled after the work of Glanz et al. (1994). Participants were asked to select an option that best reflected their intention to wash their hands for 20 s multiple times per day. Response options included “I do not intend to do this,” “I have thought about doing this, but do not yet plan to,” “I intend to do this, but have not done it yet,” “I am actively doing this,” and “This is something that I have done for a long time, and intend to continue doing to prevent disease.”

### Procedure

The present study was conducted as part of a larger project on COVID-19 and was approved by the University of Toledo Institutional Review Board. All procedures were conducted in compliance with the guidelines of the American Psychological Association. Participants were eligible for inclusion if they were at least 18 years old and resided in the United States. Before responding to the measures used in the present study, participants first were shown one of five brief handwashing messages for other purposes. The dependent variables in the present study, COVID-19 related handwashing behavior change, and readiness to change, did not differ based on the message shown. Only handwashing intentions were impacted by this message manipulation, and specifically, only one group differed from the other four. This group was excluded from the analysis of behavioral intentions to ensure that the message manipulation did not impact the present findings. It should be noted that the analyses produce similar results when they are conducted with all participants by controlling for message condition. Participants then completed the previously described measures on their attitudes, affect, intentions, and readiness to change related to handwashing. Finally, participants concluded their participation by completing demographic items.

## Results

Table 1 provides the means, standard deviations, and bivariate correlations for the measures of handwashing attitudes, affect, intentions, behavior, and readiness to change. Notably, affect and attitudes were both positively correlated with COVID-19 related change in handwashing behavior, intentions, and readiness to change.

**Table 1:** Means, standard deviations, and correlations of the variables.

		*N*	*M*	*SD*	1	2	3	4	5	6
1.	Attitudes	344	6.67	0.50	-					
2.	Affective responses	344	5.47	1.07	0.39^***^					
3.	Previous handwashing	343	6.43	6.77	0.04	0.23^***^				
4.	Current handwashing	343	10.93	8.35	0.13^**^	0.30^***^	0.79^***^			
5.	Intentions	276	4.40	0.62	0.41^***^	0.48^***^	0.21^***^	0.30^***^		
6.	Readiness to change	341	4.25	0.77	0.22^***^	0.33^***^	0.25^***^	0.25^***^	0.47^***^	
7.	Income	342	6.44	3.30	−0.01	−0.01	0.01	0.05	−0.01	0.02

** *p* < 0.01;

*** *p* < 0.001. The means and standard deviations of the handwashing behavior variables represent the raw, untransformed variables.

Given the correlations between variables, relative weights analyses (RWA; Johnson, 2000; Tonidandel et al., 2009) were used to determine the relative importance of attitudes and affect in explaining their relationship with the dependent variables of behavior change, behavioral intentions, and readiness to change. In RWA, predictor variables are transformed into orthogonal variables that are maximally related to the original predictors to determine the amount each predictor contributes to the total predicted variance and considers a predictor’s direct effect (Johnson and LeBreton, 2004). The results from each analysis is described below and a summary of the findings can be found in Table 2.

**Table 2:** Relative weights analysis examining the association of attitudes and affect with handwashing behavior, intentions, and readiness to change.

Variable	*b*	*β*	*RW*	*CI-L*	*CI-U*	*RS-RW*	*R*^2^
**Handwashing in response to COVID-19**							
Handwashing attitudes	0.10	0.08	0.011	−0.002	0.035	1.663	
Handwashing affect^*^	0.05	0.08	0.041	0.013	0.080	6.356	
Prior handwashing^*^	0.72	0.77	0.589	0.505	0.661	91.982	
**Behavioral intentions**							
Handwashing attitudes^*^	0.31	0.27	0.114	0.036	0.194	38.320	
Handwashing affect^*^	0.22	0.39	0.184	0.086	0.278	61.680	
**Readiness to change**							
Handwashing attitudes	0.18	0.11	0.031	−0.001	0.078	25.795	
Handwashing affect^*^	0.21	0.28	0.088	0.028	0.170	74.205	

* indicates statistical significance determined by 95% CI.

*b* and *β* represent unstandardized and standardized regression coefficients, respectively. *RW*, raw relative weight; *CI-L*, lower bound of confidence interval used to test significance of *RW*; *CI-U*, upper bound of confidence interval used to test *RW*; *RS-RW*, rescaled relative weight shown as the percent of predicted variance in the outcome variable attributed to each predictor variable. Handwashing behavior *n* = 342; Behavioral intentions *n* = 277; readiness to change *n* = 344.

### COVID-19 Handwashing Behavior Change

Changes in the number of times per day participants washed their hands before and after knowledge of COVID-19 was first assessed. Both the pre- and post-COVID-19 handwashing variables displayed considerable positive skew. As such, a natural log transformation was applied to both variables. Analyses were run both pre- and post-transformation. Both results were significant and produced similar findings. The transformed variable was used in the presented analyses. In an RWA, when controlling for handwashing behavior prior to COVID-19, affect, but not attitudes was found to be a significant predictor of COVID-19-related handwashing behaviors.

### Handwashing Behavioral Intentions

Mirroring the first RWA, analyses were also run with behavioral intentions as the outcome variable. The results suggest that handwashing attitudes and affect are distinct significant predictors of handwashing intentions, with affect emerging as the stronger of the two predictors.

### Readiness to Change

Again, similar to the previous analyses, an RWA was used to determine the relationship between handwashing attitudes, affect, and readiness to change. The RWA indicated that, similar to handwashing behavior, only affect was a significant predictor of readiness to change.

### Income

**Figure 1:**
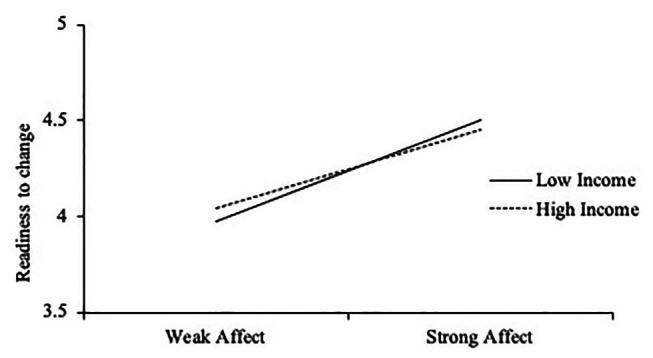
Income level moderates the relationship between affect and readiness to change.

Next, moderation analyses were conducted using the PROCESS macro for SPSS (Hayes, 2017) to determine if income moderated the relationship between attitudes and affect, and handwashing behaviors, intentions, and readiness to change. Analyses revealed income to be a significant moderator (*b* = −03, *SE* = 0.01, *p* = 0.006) of the relationship between affect and readiness to change, *F*(3, 335) = 7.71, *p* < 0.001, *R*^2^*_change_* = 0.02. Specifically, stronger affective responses resulted in greater readiness to change for low-income Americans, but not high-income Americans (see Figure 1). Income did not moderate the effects on behavioral intentions or handwashing behavior.

## Discussion

The COVID-19 pandemic is the first of its kind occurring in modern times. As such there is limited data available to understand and make sense of the way that individuals respond to and react during a time of disease-related crisis. Gaining an understanding of public behavior provides the opportunity to better intervene at the preventative level, allowing for proven biomedical and epidemiological strategies to be more widely utilized and adopted. Behavioral research focused on COVID-19 is therefore critical in order to empower governments and health care organizations to intervene in the current pandemic and prepare for future events.

The present study begins to disentangle the relationships between both cognitive and affective variables on key health behaviors at an early stage of a rapid public health crisis. The RWAs considering the relative weights of attitudes and affect in predicting COVID-19 handwashing and readiness to change revealed handwashing affect to be a relatively stronger predictor of actual handwashing than handwashing attitudes. This advantage of affect over attitudes was more pronounced on the measure of readiness to change handwashing behavior. These findings add to emerging affective science database which indicates that cognitive variables, such as attitudes, are typically better predictors of behavioral intentions, and that affective variables are more closely related to the actual behavior (Williams et al., 2018). These findings highlight the importance of considering the affective impact of interventions, as affective variables may be the driving factor behind actual behavior change and readiness to make those changes.

Despite this finding, cognitive variables should not be dismissed. Actionable interventions for behavior change often necessarily focus on changing participant intentions, which are often directly predictive of behavior. The present study provides evidence for the unique impact of both cognitive and affective variables on behavioral intentions in the context of a pandemic, and highlights the importance of targeting both variables for intervention. As predicted, attitudes toward handwashing were found to be significant predictors of handwashing behavioral intentions. Also, in support of our hypotheses, affective responses were found to better predict handwashing behavioral intentions beyond the impact of attitudes, in that the relative importance of affect was stronger than that of attitudes. This lends support to past studies demonstrating that affective constructs are distinct from cognitive variables, and additionally shows that this is true even during times of increased attention to health due to an abundance of health-related messaging and high personal relevance. Importantly, these data suggest that handwashing interventions should target both attitudes and affect.

Beyond these findings, it was also demonstrated that household income significantly interacted with handwashing affective responses when predicting readiness to change, but not when predicting past behavior change or behavioral intentions. Low-income individuals showed lower readiness to change than high-income individuals when they had weak affective responses; however, their readiness to change was higher than high-income individuals when they had strong affective responses. One possible explanation for this moderation occurring for readiness to change, but not COVID-19 related behavior change or intention, may be that while participants of all income levels display similar intentions to change, temporal readiness to change, as captured in participants’ stage of change, may capture forecasts of future internal and external barriers that impede enaction of this intention, and strong affective responses may work to overcome these barriers.

### Limitations and Suggestions for Future Research

The current results should be understood in light of the study’s limitations. The present data is cross-sectional and self-reporting in nature. The behavior change variable utilized in the present study was self-report in nature and future research should include other measurements. This variable also addresses past behavior, as opposed to future behavior change, which does not allow for the causal influence of intentions on the behavior to be explored. Relatedly, the measure of attitudes used in the present study was limited to two items due to the need for brevity. While care was taken to use items that would capture participants’ attitudes, it is possible that results may be biased by having a more robust, 5-item measure of affect. Relatedly, income was employed as a moderator due to its established connection to health behavior and perceptions, as well as to emerging data that it is a risk factor for becoming seriously ill from COVID-19. Income, however, is an overarching societal-level variable, like age and education, that can be linked to many specific psychological processes. As such, additional work is needed to clarify the important moderating effect derived from income.

Given the promising present results, however, future studies should also aim to determine how other cognitive variables, such as perceived susceptibility, subjective norms, and perceived control impact handwashing intentions, readiness to change, and behavior.

While the present study determined that income level moderates the relationship between affect and readiness to change, future studies should consider the influence of affect and attitudes as mediators of the demographic predictors of COVID-19 preventative behaviors. Future work should also aim to differentiate the impact of positive and negative affective responses, as differences can manifest in their predictive ability (Geers et al., 2017), and should also consider differences in self-conscious and hedonic affective states. For example, while not a primary focus of this study, both self-conscious and hedonic affective states were included in the measure of affect used here. *Post hoc* exploratory analyses found that these states significantly differed from one another, with hedonic states being rated as stronger. Upon examination, it appears that these types of affect were also distinct predictors of intentions, while only hedonic emotions were predictors of readiness to change. These results are in line with the findings of Giner-Sorolla’s (2001) findings that hedonic affect was more accessible than self-conscious affect in situations related to self-control. Since these analyses were *post hoc*, future studies are needed to explore the impact of different types of affective states on health behaviors.

### Conclusion

While extensive research has been devoted to understanding variables related to preventative health behaviors, little is known about how these variables perform in the United States during a pandemic, such as COVID-19. The present results provide initial evidence that cognitive and affective variables are distinct predictors of behavioral intentions and demonstrates the moderating role of household income in predicting readiness to change. This suggests the promise of interventions targeting both attitudes and affect for increasing handwashing behaviors and suggests that these types of interventions may be particularly efficacious in low-income communities.

## Data Availability Statement

The datasets presented in this study can be found in online repositories. The names of the repository/repositories and accession number(s) can be found at: osf.io/bdx8g.

## Ethics Statement

The studies involving human participants were reviewed and approved by University of Toledo Institutional Review Board. The patients/participants provided their written informed consent to participate in this study.

## Author Contributions

KC was the lead writer of the original draft, involved in the conceptualization and developing the methodology of the project, and responsible for the collection and formal analysis of the data and visualizing data. JM was involved in the conceptualization of the project and the interpretation of results, and contributed to the original draft and review and editing. KF was involved in the conceptualization and methodology of the project and editing and revision process, aided in the development of project materials, and provided supervision. AG was involved in the supervision, conceptualization, and methodology of the project, aided in the development of project materials and formal data analysis, contributed to the original draft, and provided editing and revisions. All authors contributed to the article and approved the submitted version.

### Conflict of Interest

The authors declare that the research was conducted in the absence of any commercial or financial relationships that could be construed as a potential conflict of interest.
